# Vacuum assisted closure for defects of the abdominal wall after intestinal transplantation

**DOI:** 10.3389/frtra.2022.1025071

**Published:** 2022-12-06

**Authors:** Rafael S. Pinheiro, Wellington Andraus, Allana C. Fortunato, Flavio H. F. Galvão, Lucas S. Nacif, Daniel R. Waisberg, Rubens M. Arantes, Andre D. Lee, Vinicius Rocha-Santos, Rodrigo B. Martino, Liliana Ducatti, Luciana Bertocco de Paiva Haddad, Regis O. F. Bezerra, Luiz Augusto Carneiro-D'Albuquerque

**Affiliations:** ^1^Disciplina de Transplante de Figado e Orgaos do Aparelho Digestivo, Departamento de Gastroenterologia, Faculdade de Medicina, Hospital das Clinicas HCFMUSP, Universidade de São Paulo, São Paulo, SP, Brazil; ^2^Departamento de Radiologia, Faculdade de Medicina, Instituto do Cancer do Estado de São Paulo, Universidade de São Paulo, São Paulo, SP, Brazil

**Keywords:** transplantation, intestines, short bowel syndrome, abdominal wall, negative-pressure wound therapy, hernia, abdominal

## Abstract

**Background:**

Isolated intestinal transplantation (IT) is indicated in cases of intestinal failure (IF) in the absence of severe liver dysfunction. Short bowel syndrome (SBS) is the most frequent IF etiology, and due to the absence or considerable reduction of intestinal loops in the abdominal cavity in these patients, there is atrophy and muscle retraction of the abdominal wall, leading to loss of the abdominal domain and elasticity and preventing the primary closure of the abdominal wall. This study aimed to describe a technique for the closure of the abdominal wall after IT without using prostheses.

**Methods:**

Four patients underwent IT with the impossibility of primary closure of the abdominal wall. We describe a novel technique, associating a series of vacuum-assisted closure dressings, components separation, and relaxation incisions.

**Results:**

All patients presented a successful closure of the abdominal wall with the described technique, with no complications related to the abdominal wall.

**Conclusion:**

The technique proved to be safe, effective, and reproducible as an option for abdominal wall closure after IT. Employing this technique in a greater number of cases is necessary to confirm these results.

## Introduction

In intestinal transplantation (IT), the main organ to be transplanted is the small intestine, which can be performed in combination with other organs. The type of transplantation depends on the underlying disease, the quality of other abdominal organs, the presence of liver disease, and the number of previous abdominal surgical procedures. Isolated IT is indicated in cases of intestinal failure (IF) in the absence of severe liver dysfunction.

Short bowel syndrome (SBS) is the most frequent IF etiology, accounting for 35–75% of cases (1, 2). Due to the absence or considerable reduction of intestinal loops in the abdominal cavity, these patients present atrophy and muscle retraction of the abdominal wall, leading to loss of abdominal domain and elasticity. This complication is an aggravating factor in IT since it prevents the primary closure of the abdominal wall. Moreover, graft edema after revascularization tends to worsen the difficulty in achieving tension-free primary closure (3). A forced attempt of closing the wall can result in compartment syndrome and ischemia with consequent graft necrosis and high morbimortality (4).

Several techniques for attempting to solve the problem of abdominal wall closure have already been proposed, ranging from elementary techniques, such as component separation, to the complex technique of abdominal wall transplantation; however, all these techniques potentially present severe complications that put at risk the survival of both the graft and the patient. Successful closure in this chronically ill and highly immunosuppressed population proved to be very important to decrease the risk of infections, fistulas, and mycotic aneurysms, as well as to improve the survival of grafts and patients (5).

This study aimed to propose a technique for abdominal wall closure that is easy to perform, reproducible, and reduces complications related to the abdominal wall in patients after IT.

## Methods

Our study was carried out at the Hospital das Clínicas of the Medical School of the University of São Paulo (HC-FMUSP) by the Transplantation Division of Liver and Digestive Tract Organs, from December 2017 to February 2021.

Four patients underwent isolated IT due to SBS and their abdominal wall closure was performed in series with the novel technique described in our study. All patients presented severe reduction of the abdominal cavity with severe fibrosis and wall atrophy due to multiple previous surgical procedures and could not undergo tension-free primary closure without using a prosthesis.

The studied variables were age, gender, number of surgical procedures, days required for complete closure of the cavity, and quantification of the increase in the abdominal cavity. To quantify the progress of the cavity expansion, we performed a tomographic comparison of the measurements of both anteroposterior distance and intra-peritoneal area in the corresponding coronal section, before and after the IT, with the abdominal cavity already completely closed. The reference points for measurements were the anterior face of the vertebral body at the level of the L3 vertebra up to the umbilical scar's skin. The intra-peritoneal area was measured in cm^2^, and the difference between pre- and post-IT values was shown in percentage.

All the patients included in our study signed the informed consent form.

Our study was approved by the HC-FMUSP research ethics committee, opinion no. 3,730,175.

### Description of the technique

**Figure 1:**
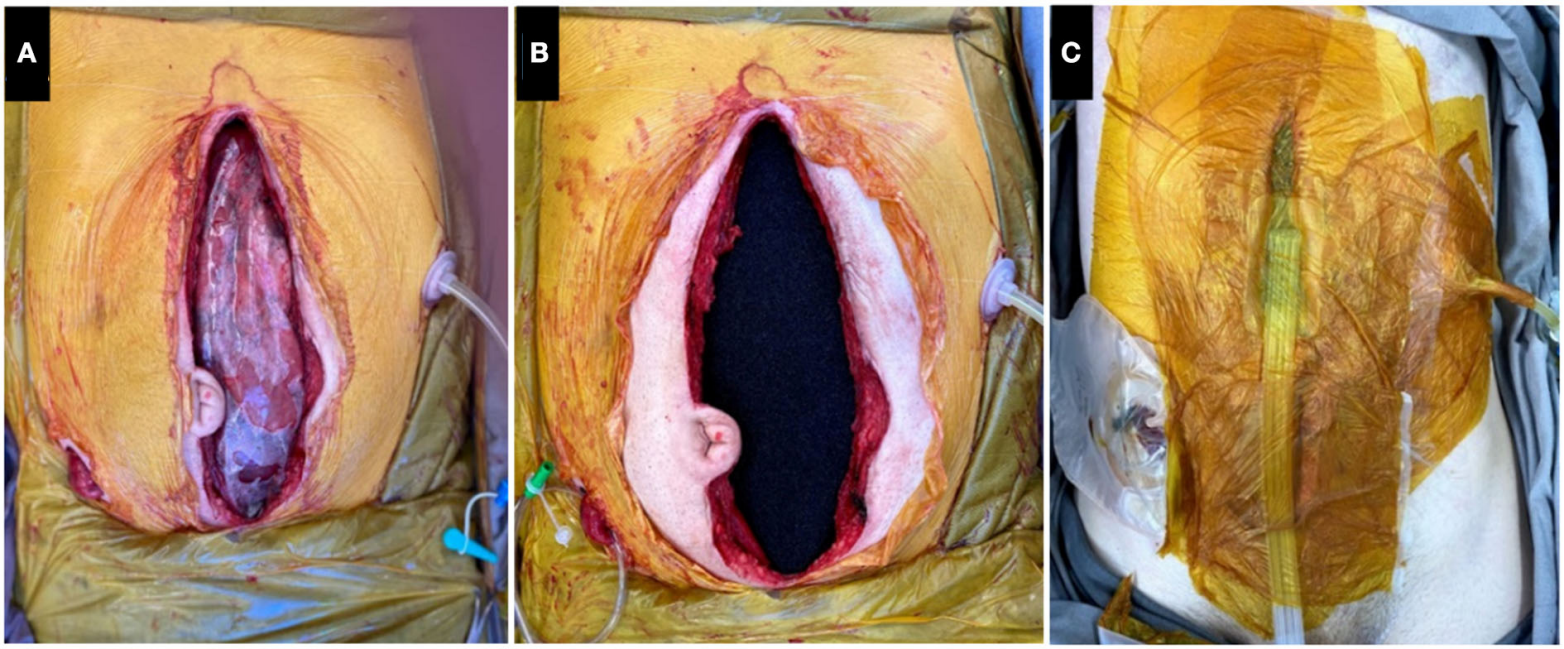
**(A)** Sterile multi-perforated plastic covering the viscera; **(B)** Polyurethane foam over the multi-perforated plastic coverage. **(C)** Vacuum created with the aid of plastic films.

A vacuum-assisted closure (VAC) dressing is applied on the transplantation day, at the end of the procedure, as follows: coverage of all abdominal viscera with a sterile multi-perforated plastic (Figure 1A); insertion of cross-linked polyurethane foam with 400 μm to 600 μm pores over the plastic coverage in order to cover the aponeurotic defect (Figure 1B); coverage of incision, foam, and adjacent skin with meticulously coupled plastic adhesive strips in order to cover external holes (gastrostomy/duodenostomy/ileostomy); positioning of the negative pressure device on the foam and connecting to the continuous vacuum pump (Figure 1C). The patient is then transferred to the ICU.

**Figure 2:**
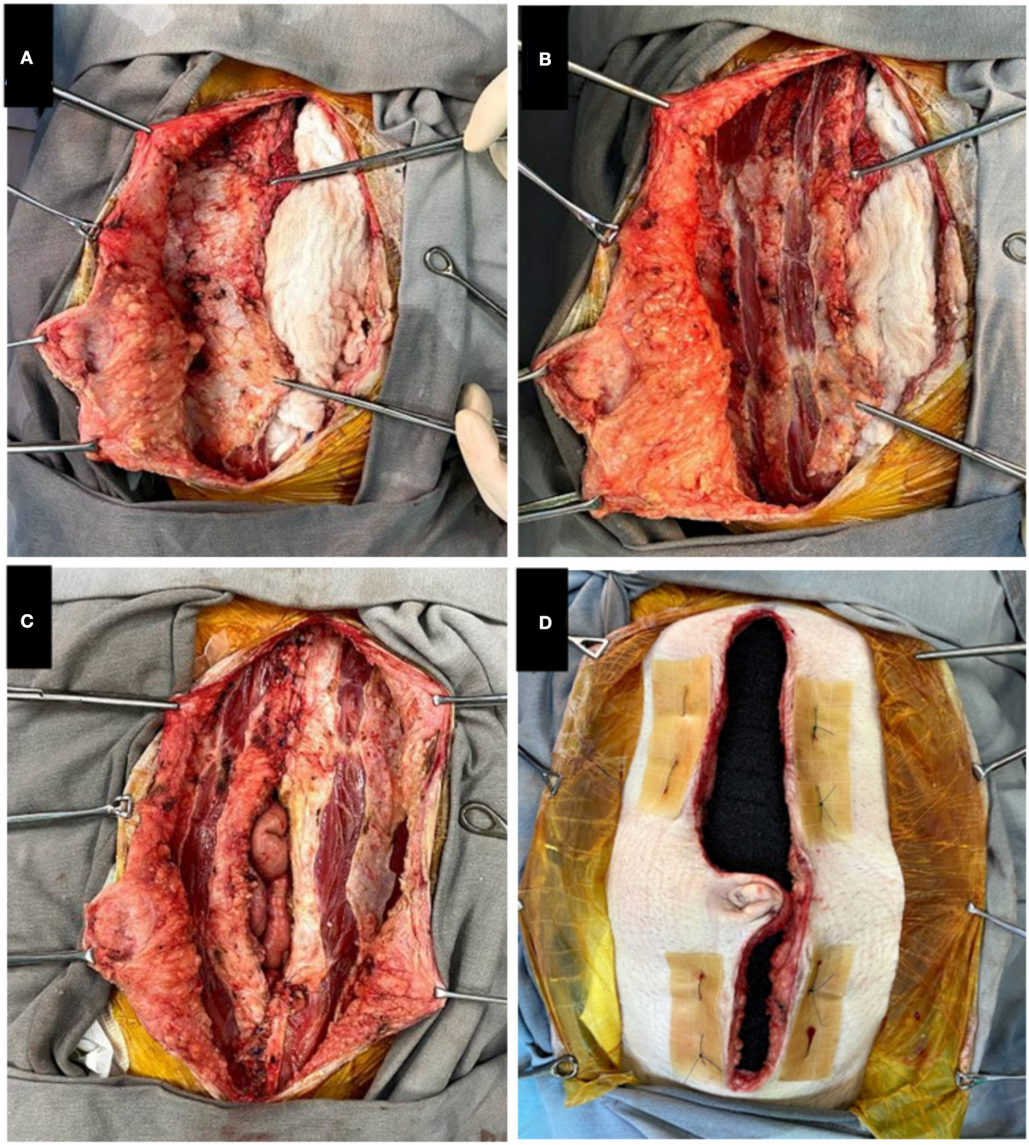
**(A)** Components separation. **(B)** Relaxation incisions. **(C)** Approximation of edges with non-absorbable stitches. **(D)** Stitches for guiding the vacuum, protected by hydrocolloid.

Within 48 to 72 h after the transplantation, a new surgical procedure is performed for sterile removal of the previous dressing. At this moment, a wide components separation of the abdominal wall is performed, freeing the anterior sheath of both the rectus abdominis and external oblique muscles bilaterally from subcutaneous fat and skin (Figure 2A). Then, relaxation incisions are made in the anterior sheath of the rectus abdominis muscle (Figure 2B). Both aponeurosis and tension-free edges of the skin are sutured with non-absorbable thread and stitches are separated (Figure 2C). In addition, some separated “U” stitches are performed on the aponeurosis and skin in order to approximate the edges and guide the vacuum force for posterior closure. To prevent lacerations, the skin is protected by hydrocolloid plaques (Figure 2D), followed by a new VAC dressing.

**Figure 3:**
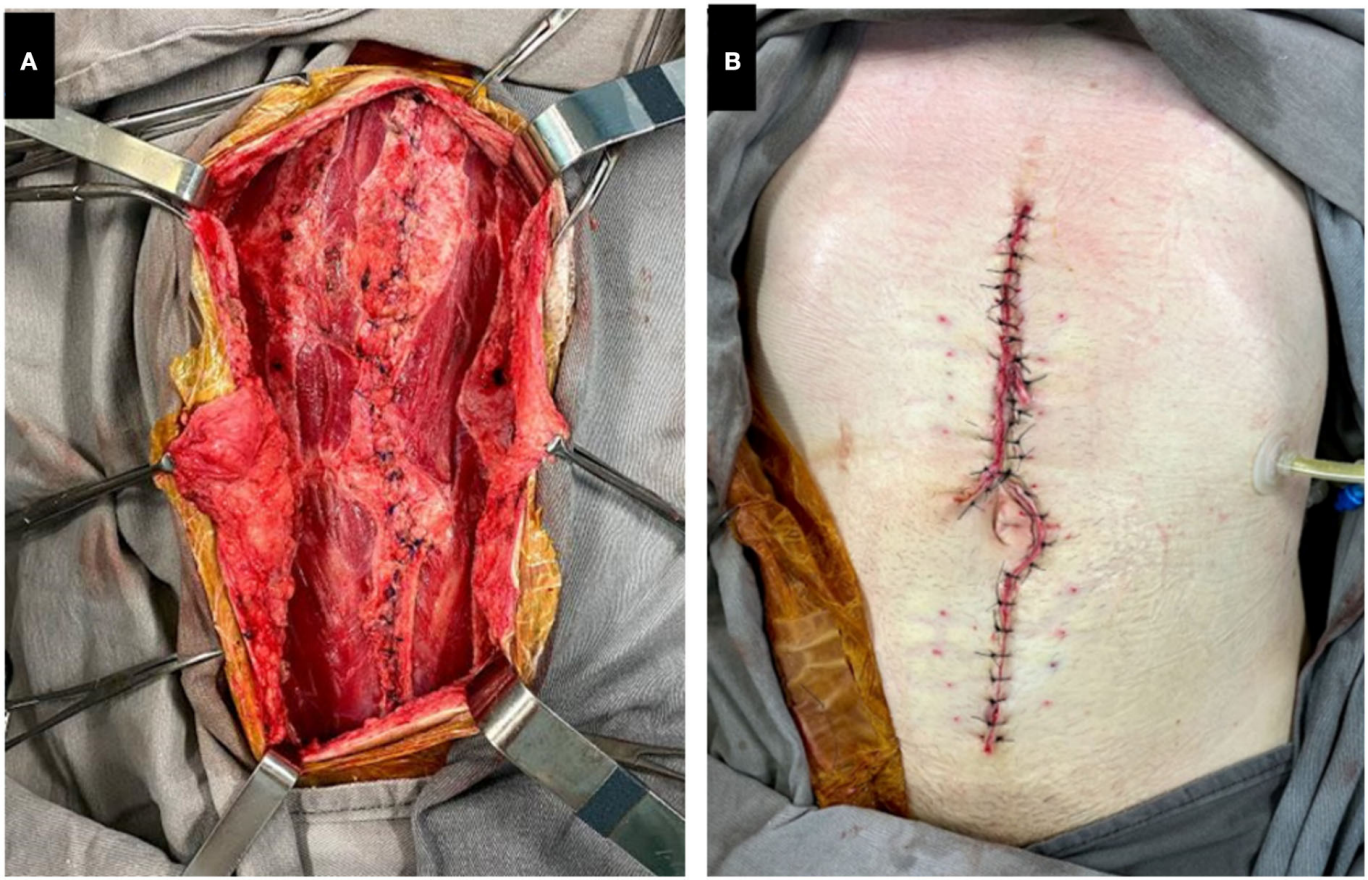
**(A)** Full closure of the aponeurotic defect. **(B)** Full closure of the skin. During the interval between surgical procedures, motor physical therapy and early ambulation are encouraged. Since the negative pressure device is mobile, the patient can easily move it around with the help of a mobile support, such as an intravenous infusion pump.

Thereafter, new procedures are carried out every 48–72 h in order to suture the tension-free edges of the skin and aponeurosis. If there is still an aponeurotic defect, new approximation stitches in both skin and aponeurosis, as well as a new vacuum dressing, are performed. This step is repeated until achieving full primary closure of the abdominal defect (Figures 3A,B). If the previous relaxation incisions are already healed, new ones are performed in different locations.

There is a recommendation for only preoperative fasting preceding the surgical procedure. During the intervals, the patient receives enteral nutrition according to the nutritional goal, without calorie and protein loss.

Six months after the transplantation, ileostomy takedown was successfully performed.

## Results

The four patients in our study were male, with ages ranging from 19 to 45 years old. The mean number of procedures for changing the dressings was 3.6 ± 2 and the mean period of reoperations per patient was 15 ± 11.2 days (Table 1). No patient presented complications related to the closure of the abdominal wall during the period of changing dressings or after complete healing.

**Table 1:** Characteristics of patients who underwent intestinal transplantation at Hospital das Clinicas of the University of São Paulo (HCFMUSP).

	**Patient #1**	**Patient #2**	**Patient #3**	**Patient #4**
Age (years)	25	45	27	21
Gender	Male	Male	Male	Male
Weight (kg)	60	55	70	51
BMI	19,6	18	18	19,4
Donor age (years)	14	19	30	5
Donor weight (kg)	60	75	70	26
Donor BMI	20.7	25.9	24.2	20.3
Time of IF (months)	24	12	96	72
Right colon inclusion in the graft	NO	YES	YES	YES
Inferior caval thrombosis	YES	NO	NO	NO
Total number of VAC changes	3	6	2	9
Days from IT to complete AW closure	8	28	9	21
Wound infection	NO	NO	NO	NO
Dehiscence	NO	NO	NO	NO
Abdominal volume before IT (cm^3^)	2.681,59	5.072,92	3.681,8	4.339,77
Abdominal volume after IT (cm^3^)	4.562,56	5.994,76	6.395,56	5.369,06
Increase in abdominal volume	170%	118%	173%	123%
AW hernias	NO	NO	NO	NO

BMI, body mass index; IF, intestinal failure; IT, intestinal transplantation; AWC, abdominal wall closure.

In all cases, immunosuppression was induced with 1.5 mg/kg thymoglobulin (ATG) and the tacrolimus started on the first postoperative day by jejunostomy. According to the institution's protocol, patients also received Rituximab on the third postoperative day. Mycophenolate mofetil (MMF) was introduced on the fifth postoperative day. All patients kept triple immunosuppression for at least 6 months.

**Figure 4:**
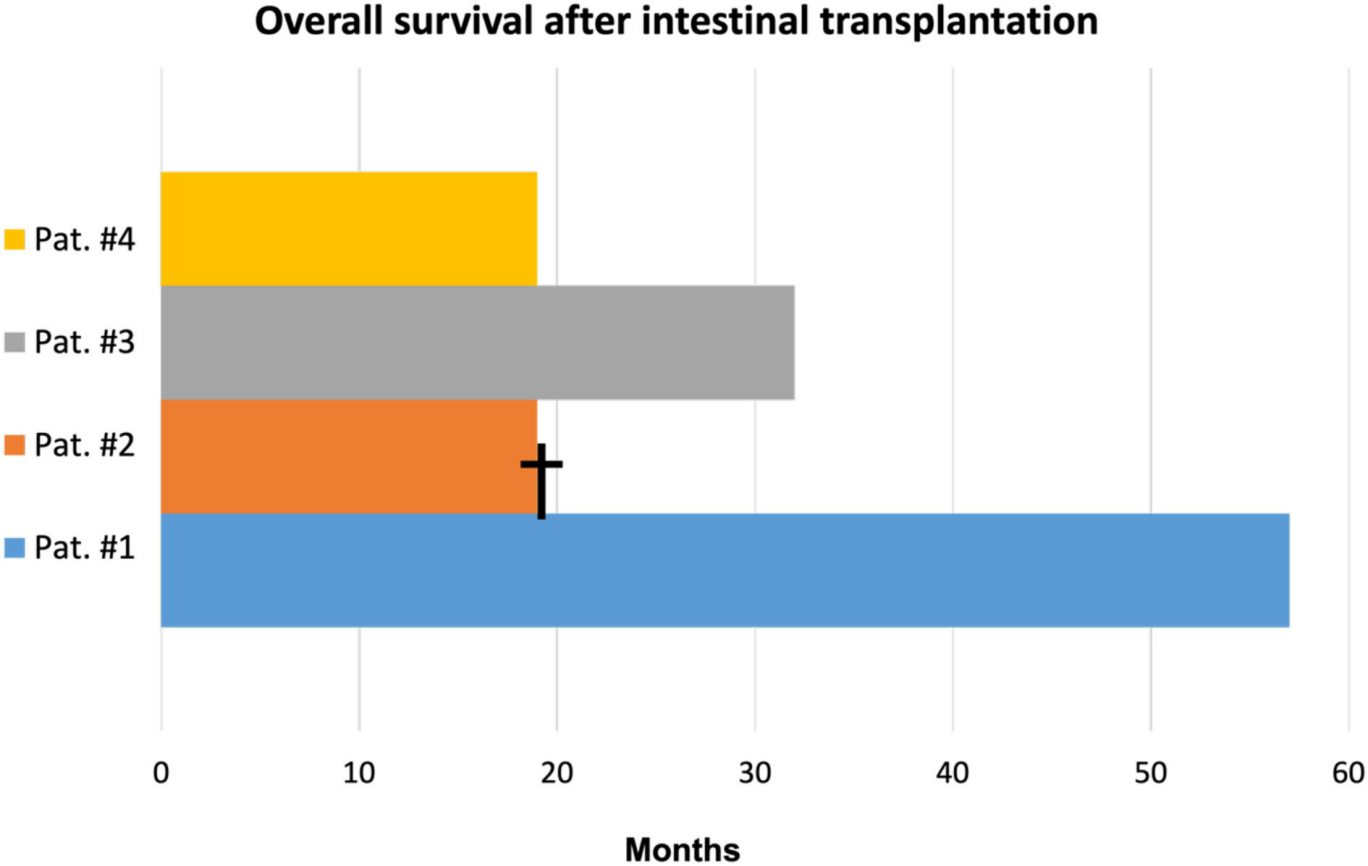
Overall survival of patients undergoing Intestinal transplantation at HC-FMUSP. †death.

The graft and patient survival rate was 100% in 18 months (Figure 4). No patient developed hernias, dehiscence, surgical site infection, or any other complications related to the abdominal wall. Despite the great detachment promoted by the separation of the components, no patient evolved with seroma or subcutaneous collections after complete closure and vacuum removal.

In all cases, the diet was reintroduced before the complete closure of the abdominal wall. All patients received an early postoperative diet and were stimulated with motor physical therapy and ambulation during the interval between surgical procedures. Ileostomy takedown was performed 6 months after the complete closure of the abdominal wall. In this procedure, adhesions between the intestinal loops and the abdominal wall were not observed.

**Figure 5:**
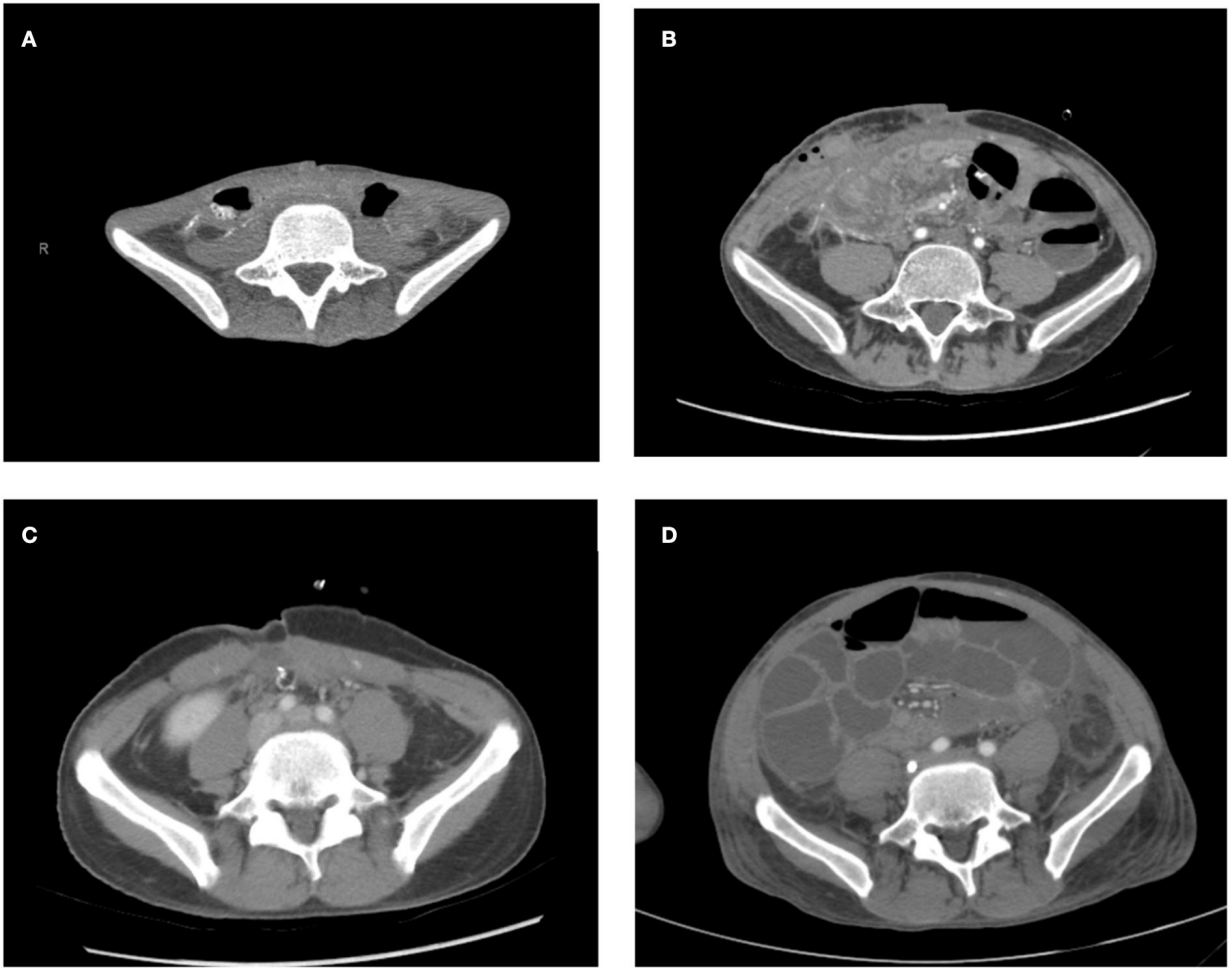
**(A,C)** CT before IT. **(B,D)** CT after IT, showing a significant increase of the abdominal area.

Computed tomographies demonstrated a significant gain in the abdominal domain after transplantation in comparison to pre-IT scans (Figures 5A–D). The patients' pre-transplantation median of anteroposterior distance was 63.2 mm (min: 29 mm and max: 73.3 mm), and the median after abdominal wall closure was 98.8 mm (min: 90 mm and max: 124.4 mm), showing a considerable increase.

**Figure 6:**
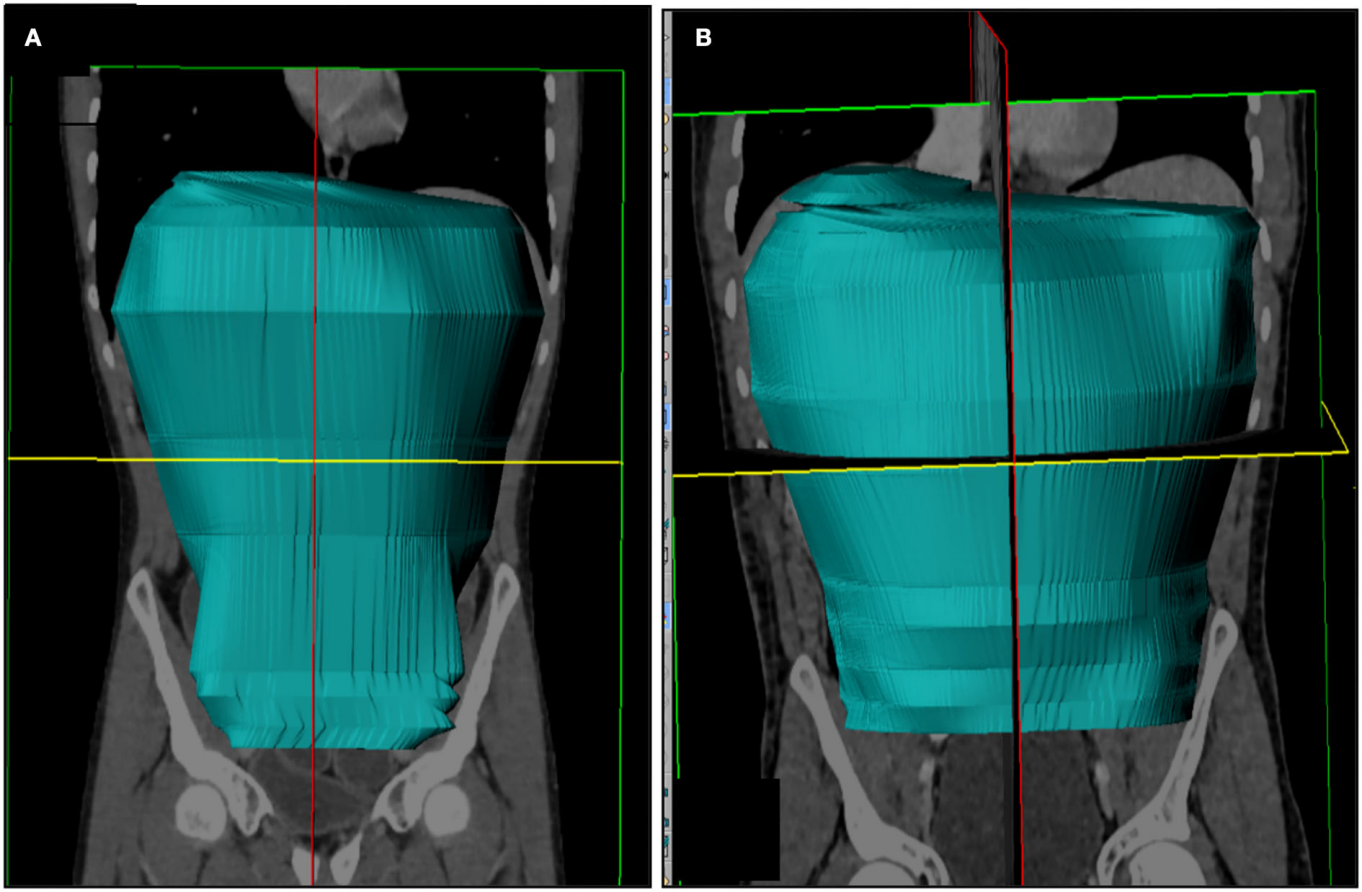
Radiological evaluation of abdominal volume before **(A)** and after **(B)** intestinal transplantation.

Regarding the abdominal cavity area, the pre-operative median volume was 4,010.7 cm^3^ (min: 2,681.5 cm^3^ and max: 5,072.9 cm^3^) and post-operative was 5,681.9 cm^3^ (min: 4,562.5 cm^3^ and max: 6,395.5 cm^3^). Therefore, in the corresponding coronal area, postoperative volume increase had a median of 1,455.13 cm^3^ (min: 921.84 cm^3^ and max: 2,713.76 cm^3^), representing a 41.49% increase (Figures 6A,B).

## Discussion

The technique proposed by us in our study is innovative, safe, and has the potential to be reproducible in most patients undergoing IT. It has three important benefits: resolving defects in both skin and aponeurosis, dismissing the use of prosthetic meshes, and performing efficient drainage of the abdomen and subcutaneous tissue, thus reducing the risk of collections.

When primary closure is not possible, there are several strategies that have been described for reconstructing the abdominal wall after an IT. The main approaches focus on reducing the volume of the graft or expanding the abdominal cavity (4). Reducing the volume of the graft can be achieved using small donors (6) or anatomical reduction of the graft's size through bench surgery (7), mainly in pediatric transplants, in order to avoid high mortality rates among those on the waiting list (8). However, reduced-size grafts are related to high rates of complications. In addition, due to a high scarcity of organs, the use of small donors in adults is less frequent. Thus, techniques for increasing the abdominal cavity are preferred.

Techniques vary in complexity; use of prostheses and postoperative morbidity. Techniques such as components separation, use of absorbable meshes, use of non-absorbable meshes, use of tissue-engineered skin equivalents, acellular dermal matrix, skin grafts, and transplantation of the abdominal wall (9) and of the aponeurosis, have been described.

The closure of the abdominal wall after graft implantation in the IT presents some aggravating factors since many patients have a chronic reduction of the abdominal cavity. In patients with successful primary closure after IT, about 20% to 33% progress with secondary dehiscence of the surgical wound, leading to complications such as eviscerations, fistulas, and infections (10–12). The existing techniques for abdominal wall closure involve prostheses made of synthetic or biological materials, which not only generate high costs but also increase the risk of complications. This is specifically because the mesh is in direct contact with the loops, increasing the risk of adhesions and fistulas. Moreover, in the context of severe immunosuppression and ileostomy close to the surgical wound (since the abdominal wall is reduced), the use of a prosthetic mesh provides an additional risk.

Abdominal wall transplantation, as an alternative technique for abdominal wall closure after IT, increases the complexity of the procedure, extending the time of intestinal graft ischemia (13). This graft must be vascularized with both arterial and venous epigastric anastomoses, which are at risk of complications (14). Subsequently, the technique was modified by Starzl et al. (15), using a microsurgical technique that seems to minimize vascular complications by using the donor's epigastric pedicles anastomosed directly with the recipient's epigastric vessels. Theoretically, abdominal wall transplantation imposes higher immunological risk, but some reports observed a low rate of abdominal wall rejection and prompt response to the minimal and temporary amount of steroids in cases of rejection (15). The isolated aponeurosis transplantation has an increased risk of failure due to the lower vascularization of this type of graft (4). Moreover, both the isolated aponeurosis transplantation and the use of prosthetic mesh do not solve the problem of skin closure.

Another complicating factor is the potent immunosuppression used by IT recipients. Almost all immunosuppressors cause varying degrees of impairment of wound healing, resulting in a potentially increased risk of wound dehiscence.

Corticosteroids have a negative impact on wound healing, especially when a higher dose is ordered to contrast an acute rejection (16, 17). In addition, the most used immunosuppressive scheme based on tacrolimus combined with MMF directly interferes in the wound healing process due to its antiproliferative effect caused by the reduction of fibroblasts and endothelial cell growth factors (16–21), although some authors have suggested that MMF could have a protective role (22). All mTOR inhibitors, such as everolimus, suppress skin-resident T-cells contributing to delayed wound closure. Adverse wound healing events can increase by up to 35% in patients who received everolimus (23).

Therefore, there are many techniques available in order to expand the remaining abdominal domain or replace the damaged abdominal wall in the setting of intestinal transplantation. The results obtained with these techniques were extensively debated in a recent review (24), which concluded that there is no consensus among transplantation centers regarding which technique would be ideal with higher success rates and lower rates of complications.

The vacuum-assisted closure technique for abdominal wall closure is used for critically ill patients, particularly for those with complex surgical procedures, such as trauma victims, who have undergone damage control surgery, patients with severe intra-abdominal infections, acute mesenteric ischemia, abdominal compartment syndrome, and necrotic infections of the abdominal wall (16).

In this VAC technique, the interaction of foam with the tissue under negative pressure results in macro and micro deformations that stimulate wound granulation and increase the production of pro-angiogenic growth factor, accelerating the healing (25). In addition, it contributes to the reduction of systemic inflammation and damage to abdominal organs, decreases bacterial colonization, and improves collagen deposition in the wound (26, 27). In 2001, it was evidenced that dressing under negative pressure causes a centripetal force that results in the approximation of the wound edges (28). Cheatham et al. (29) concluded that the VAC technique is associated with high rates of abdominal closure in 30 days, as well as with lower morbimortality. VAC reduces the need for medical and nursing care, especially in patients who have an excessive amount of abdominal secretion. Studies with the level of evidence and grading of recommendations 1A (30) and 2A (31) suggest dressing changes every 48 to 72 h, providing significant comfort not only to patients but also to the healthcare teams. An additional benefit is the continuous suction of the abdominal cavity, which prevents the formation of intra-abdominal collections. These collections frequently irritate the diaphragmatic muscles and can cause pleural effusions and pain. Thus, the patient may present a faster and more effective respiratory and motor recovery.

The technique proposed in our study consists of a combination of VAC with components separation and a series of relaxation incisions. It is an easily reproducible technique that does not use synthetic prostheses or biological materials, allowing the assessment of the graft at each reoperation. As a result, there is less chance of adhesions among intestinal loops and the loops with the abdominal wall, and there is the optimization of drainage of the abdominal cavity in the first postoperative days. Changing the VAC dressing does not interfere with intestinal peristalsis, thus not preventing the reintroduction of an enteral diet immediately after the procedure. During intervals between procedures, ambulation is possible without difficulty since the negative pressure device is mobile and easily carried.

The vacuum's fistulization potential is a possible criticism regarding the use of the proposed technique. This risk would be even greater in the first postoperative days since the anastomoses are still fresh and not yet healed. However, several publications have evidenced the advantages of VAC therapy in abdomens that present an impossibility of primary closure, emphasizing the low risk of developing intestinal fistula. The presence of peri-anastomosis secretion probably causes inflammation, or possible infectious focus, that is more related to the anastomosis fistula than to the negative pressure applied by the vacuum. In a series of 112 patients, only five (4.5%) developed fistula: one pancreatic, one gastric, and three in the small bowel (32). In 2005, Bovill et al. (33) evidenced that VAC is a satisfactory technique for the temporary closure of the abdominal cavity, being related to the occurrence of complications, such as fistulas, in only 2.6% of cases. Rao et al. (34) evaluated 29 patients with abdominal closure with VAC, in which 14 of these patients had intestinal anastomosis and 15 did not. Three of the patients with anastomosis developed intestinal fistulas, although the location of the fistula was different from the location of the suture. In the remaining patients without intestinal sutures, three also developed enteric fistulas. Thus, the study suggests that continuous negative pressure does not predispose the patient to the occurrence of anastomosis fistula (34). A systematic review and meta-analysis published by Atema et al. (35) identified 74 studies describing 78 series of patients, comprising 4,358 patients in total; 3,461 (79%) of them had an impossibility of abdominal closure due to non-traumatic causes. The highest rate of fistulas was observed in the association of abdominal closure with mesh (17.2%, CI 95% 9.3–29.5%, χ^2^ *p* = 0.012, *I*^2^ = 66%), while the association of VAC with fascial traction showed the lowest weighted rate of fistula (5.7%, CI 95% 2.2–14.1%, χ^2^ *p* = < 0.001, *I*^2^= 79%). Therefore, intra-abdominal complications resulting from VAC therapy are not frequent (36). The most reported complication rates in the literature are related to the previous comorbidities of patients and skin irritation due to the use of the adhesive (37). In addition, departments that perform a great volume of IT, such as Georgetown University, have already used VAC on the IT day as a tool to reduce initial edema after revascularization, as well as to enable the implantation of smaller meshes in a second moment, with no reports of intestinal fistulas.

One of the limitations of our study was the low number of patients. Future studies with a greater number of subjects will probably have new characteristics to evaluate and will contribute to a better assessment of the technique. In addition, not all patients will be eligible for complete closure using the technique that associates components separation with VAC; however, the resulting abdominal defect will be much smaller. The negative aspect of our technique is the need for surgical reoperations, which can prolong the hospital stay.

Our experience associating components separation, relaxation incisions, and VAC proved to be effective in four cases after 18 months of follow-up with a 100% survival rate; none of the patients presented complications related to the closure of the abdominal wall.

## Conclusion

The technique described in our study is safe, effective, and affordable, providing an additional possibility for abdominal wall closure after IT. In addition to being an easily reproducible technique, it allows a series of graft evaluations and early diagnosis of possible complications.

## Data availability statement

The original contributions presented in the study are included in the article/supplementary material, further inquiries can be directed to the corresponding author.

## Ethics statement

The studies involving human participants were reviewed and approved by CAPPESQ. The patients/participants provided their written informed consent to participate in this study.

## Author contributions

WA, RP, and AF have made substantial contributions to the conception and design, acquisition, and interpretation of data and have been involved in drafting, and revising the manuscript. FG and DW have made substantial contributions to the acquisition, analysis, and interpretation of data and have been involved in drafting, and revising the manuscript. AL, RA, and LN have made substantial contributions to the design, analysis, and interpretation of data and have been involved in revising the manuscript. VR-S and RM have made substantial contributions to the analysis and interpretation of data. LD has made substantial contributions to the acquisition and interpretation of data and has been involved in drafting the manuscript. LC-D'A and FG have been involved in revising critically for important intellectual content. LC-D'A has been involved in revising the manuscript critically for important intellectual content and has given final approval for the version to be published. All authors contributed to the article and approved the submitted version.

## Conflict of interest

The authors declare that the research was conducted in the absence of any commercial or financial relationships that could be construed as a potential conflict of interest.

## Publisher's note

All claims expressed in this article are solely those of the authors and do not necessarily represent those of their affiliated organizations, or those of the publisher, the editors and the reviewers. Any product that may be evaluated in this article, or claim that may be made by its manufacturer, is not guaranteed or endorsed by the publisher.

## Abbreviations

HC-FMUSP: Hospital das Clínicas of the Medical School of the University of São Paulo
ATG: Thymoglobulin
IF: Intestinal failure
IT: Intestinal transplantation
MMF: Mycophenolate mofetil
SBS: Short bowel syndrome
VAC: Vacuum-assisted closure (VAC).
